# Estimation of the Safe Zone in the Anterior Mandible for Implant Osteotomy: A Cone-Beam Computed Tomography (CBCT) Study

**DOI:** 10.7759/cureus.84654

**Published:** 2025-05-22

**Authors:** Mousumi Mahato, Sadananda Hota, Purnendu Bhusan, Anjana Raut, Arun Mohanty, Jaya Naikode

**Affiliations:** 1 Prosthodontics, Kalinga Institute of Medical Sciences, Kalinga Institute of Industrial Technology Deemed to be University (KIIT DU), Bhubaneswar, IND; 2 Prosthodontics, Kalinga Institute of Dental Sciences, Kalinga Institute of Industrial Technology Deemed to be University (KIIT DU), Bhubaneswar, IND; 3 Periodontology, K.M. Shah Dental College &amp; Hospital, Vadodara, IND

**Keywords:** anterior loop, genial tubercle, implant osteotomy, inferior alveolar nerve, safe zone

## Abstract

Purpose: This study aimed to estimate the safe zone in the anterior mandible relative to the genial tubercle. It also evaluated the genial tubercle’s reliability as a reference point for interforaminal measurements during implant planning.

Patients and methods: Four hundred ninety-nine cone-beam computed tomography (CBCT) Digital Imaging and Communications in Medicine (DICOM) files (250 men and 249 women) were examined with NNTViewer software (Newtom, Verona, Italy). These files were obtained from dental departments of academic institutions and private imaging centers. The distance from the genial tubercle to the anterior loop of the inferior alveolar nerve was measured, and a standardized protocol for determining safe zones was established.

Results: In men, the mean safe zone was 23.14 mm (right) and 22.07 mm (left). In women, it was 20.82 mm (right) and 19.47 mm (left). Overall, men showed wider safe zones, with more women falling within the 15-19.9 mm category, while more men had >25 mm safe zones. Linear regression indicated a strong correlation between the genial tubercle to anterior loop distance and the safe zone.

Conclusion: This study provides valuable data on anterior mandibular safe zones, highlighting gender-based differences and supporting safer implant osteotomy planning using the genial tubercle as a reference.

## Introduction

The inferior alveolar nerve (IAN), a terminal branch of the mandibular division of the trigeminal nerve (cranial nerve V), travels through the mandibular canal, supplying sensation to the mandibular teeth, alveolar bone, and surrounding soft tissues. Before it exits the mandible via the mental foramen, the IAN may take an anteriorly curved course known as the anterior loop (AL). This anatomical configuration extends beyond the mental foramen before looping back to its exit, and its presence is a significant surgical concern, particularly in the placement of dental implants in the premolar and interforaminal regions [1].

The prevalence, length, and morphology of the AL vary considerably among individuals, influenced by factors such as age, gender, dental status, and ethnicity. These anatomical differences are of critical clinical importance, as inadvertent nerve injury during implant osteotomy can lead to sensory disturbances such as paresthesia, anesthesia, or neuropathic pain, thereby affecting a patient's quality of life and satisfaction with treatment outcomes [2]. Therefore, precise identification and understanding of the AL and other anatomical landmarks are essential to minimize the risk of iatrogenic complications.

Traditional two-dimensional imaging modalities often fall short of accurately identifying the AL, prompting the use of cone-beam computed tomography (CBCT) in implant diagnostics. CBCT offers high-resolution, three-dimensional visualization of critical neurovascular structures, enabling precise surgical planning and improving patient safety [3].

One of the key challenges in anterior mandibular implant planning is the determination of a “safe zone”-a defined area free from vital anatomical structures, where implants can be safely placed [4,5]. This is particularly difficult in edentulous patients, where resorption and loss of conventional landmarks complicate orientation. In such cases, reliance on consistent internal landmarks becomes essential. The genial tubercle (GT), a midline bony prominence on the lingual surface of the mandible, offers a reliable and reproducible reference point for morphometric assessments in both dentate and edentulous patients [6].

Although several studies have investigated the AL and interforaminal anatomy across various populations [7], data correlating the AL with the GT are scarce. Moreover, very few studies have utilized this relationship to estimate a standardized safe zone for implant placement. A robust yet clinically applicable correlation between these two anatomical landmarks could greatly improve preoperative assessment and communication among clinicians, especially in challenging edentulous cases.

Thus, the present study aims to establish a reproducible anatomical relationship between the GT and the AL of the IAN using CBCT imaging, develop a standardized protocol for defining a safe implant osteotomy zone in the anterior mandible using these landmarks, and categorize these safe zones to enhance clinical decision-making and improve surgical outcomes.

Null hypothesis

There are no statistically significant differences in the dimensions of the safe zone between genders or between the right and left sides.

## Materials and methods

Source of data

Ethical approval for this multicenter study was obtained from the Institutional Ethical Committee, Kalinga Institute of Medical Sciences, KIIT University, with reference number: KIIT/KIMS/IEC/1305/2023. This study was done in compliance with the Declaration of Helsinki, U.S. Federal Policy for the Protection of Human Subjects. A total of 499 CBCT Digital Imaging and Communications in Medicine (DICOM) files (250 men and 249 women) were selected from the records of the Department of Oral Medicine and Radiology and Department of Prosthodontics and Crown & Bridge, Kalinga Institute of Dental Sciences, KIIT University, and from three different CBCT centers, all from different states and regions. The DICOM files were imported and viewed with the help of software (NNTViewer, Newtom, Verona, Italy). All participants involved were provided with consent forms in English and local language prior to inclusion in the study.

Inclusion Criteria

Patients aged 18 years and above, both dentate or edentulous patients, and patients opting for implant treatment were preferred.

Exclusion Criteria

The exclusion criteria include patients with congenital or developmental deformities, a syndrome affecting the jaws, traumatic injury to the mandible, and pathologic mandibles such as cysts and tumors.

**Figure 1:**
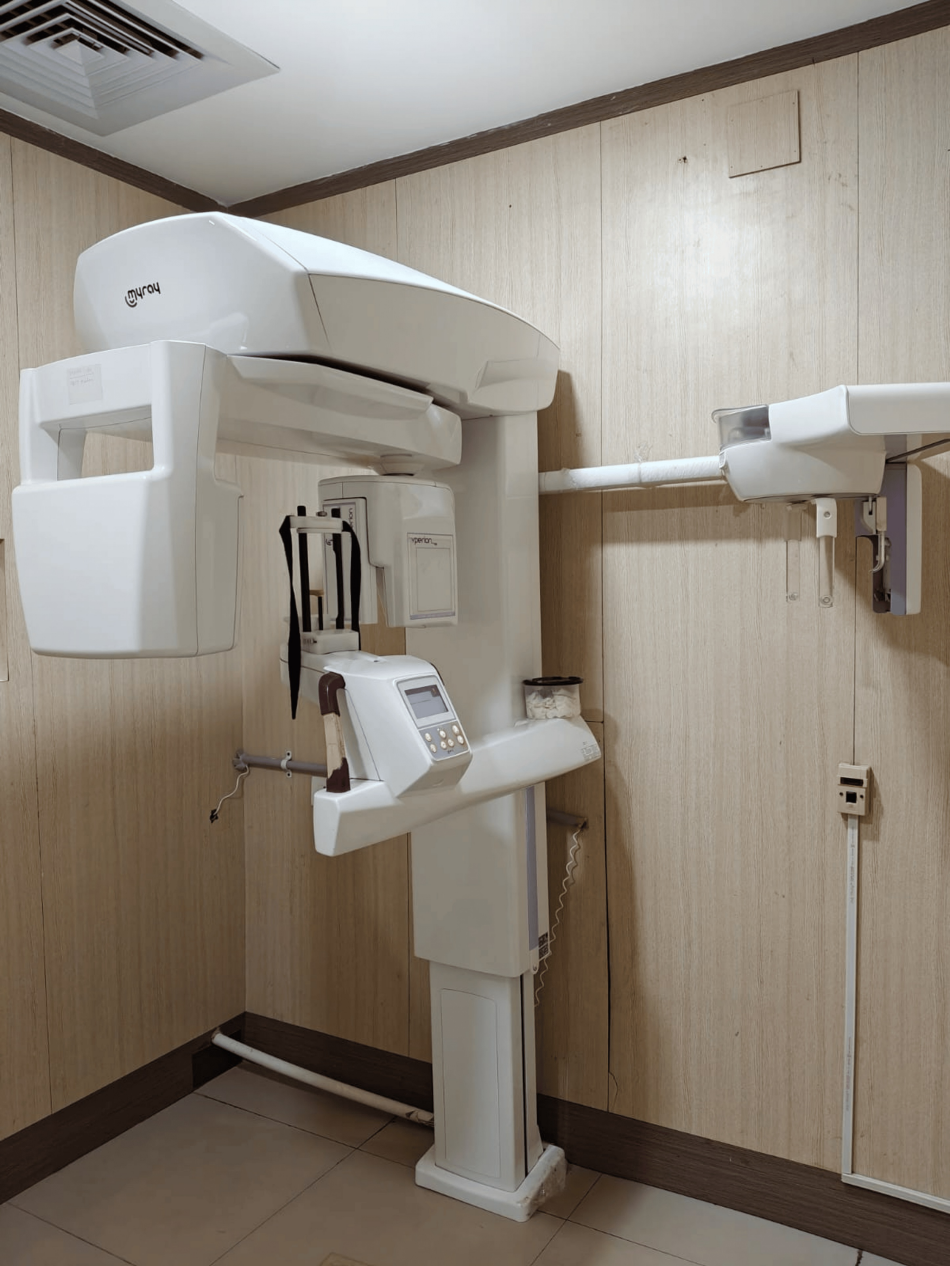
Hyperion X9 CBCT Scanner (MyRay, Charlotte, NC, US), Kalinga Institute of Dental Sciences, Kalinga Institute of Industrial Technology

The armamentarium used was the Hyperion X9 CBCT Scanner (MyRay, Charlotte, NC, US) (Figure 1), IRYS version 8.0, and NNT software.

Methodology

Estimation of the Safe Zone With the Help of GT

**Figure 2:**
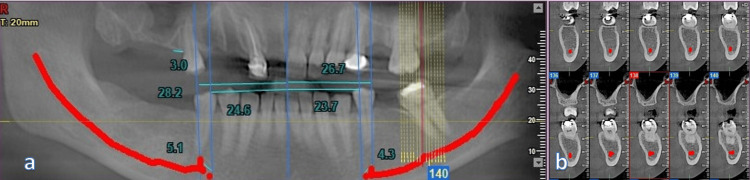
Panoramic and cross-sectional images (a) Panoramic view for evaluation of inferior alveolar nerve (IAN); (b) cross-sectional slices to determine the exact location and path of IAN

A comprehensive anatomical evaluation of the entire mandible was performed using coronal cross-sectional, horizontal, and panoramic imaging (Figure 2). Millimeter-based measurements were collected unilaterally by pinpointing the GT, the mesial border of the mental foramen, and the AL of the IAN.

**Figure 3:**
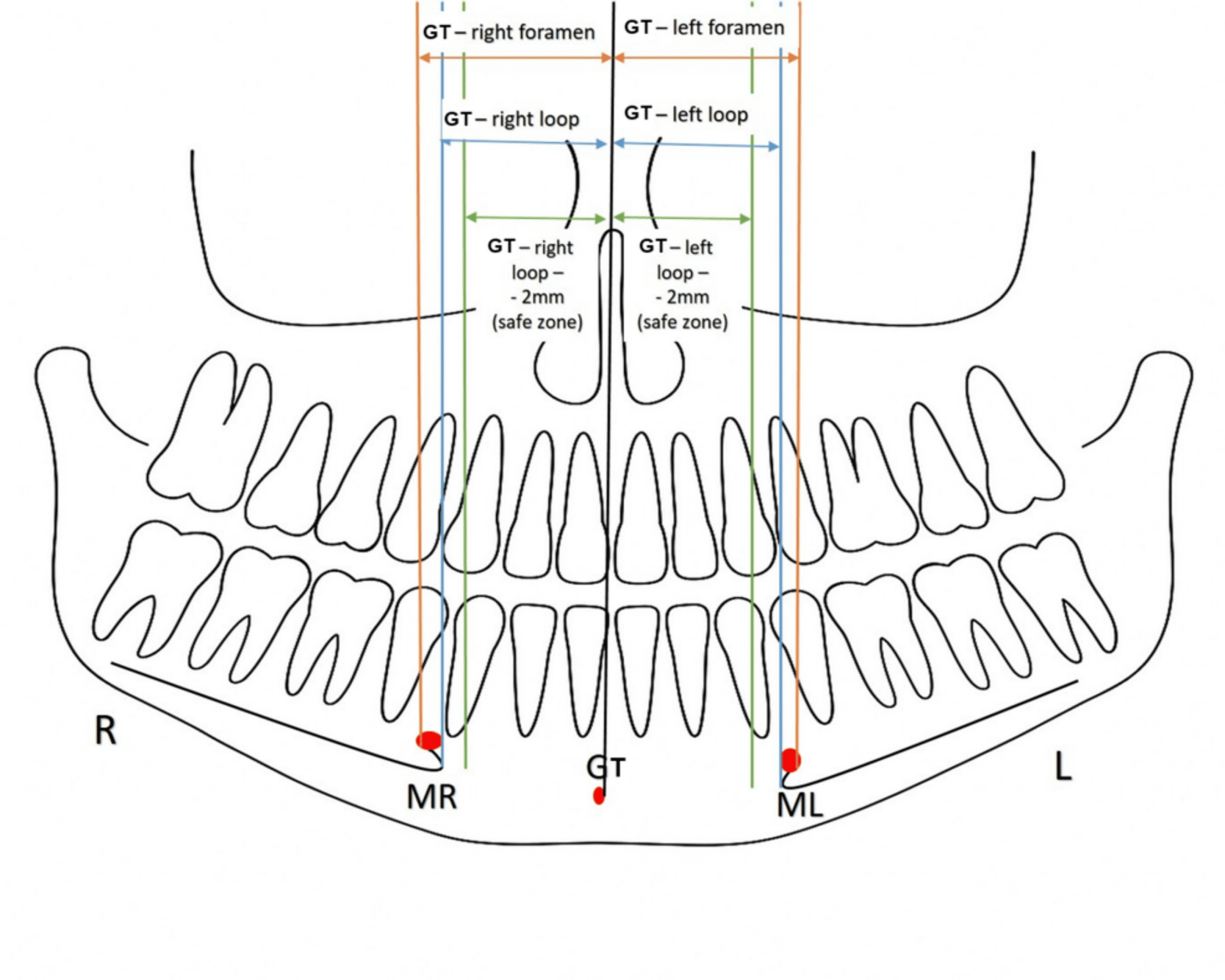
Diagrammatic representation of the various measurements from the genial tubercle (GT), mental foramen, anterior loop, and 2 mm from loop R: right side; L: left side; MR: right mental foramen; ML: left mental foramen; CBCT: cone-beam computed tomography Illustration made by tracing the outline of the panoramic view of one of the CBCTs used in the study with the help of MS PowerPoint and MS Paint editing tools (Microsoft Corp., Redmond, WA, US)

**Figure 4:**
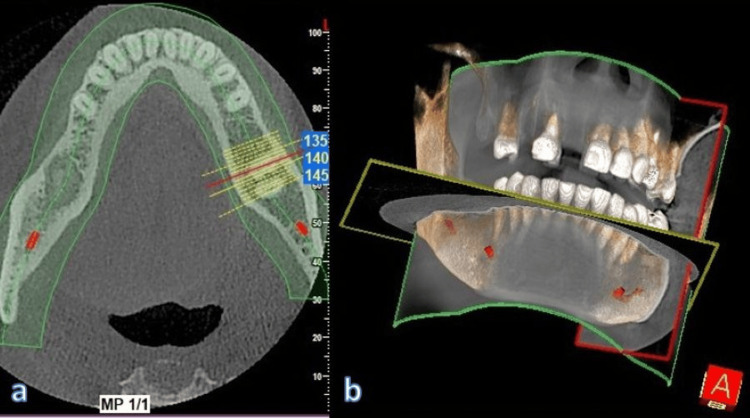
Comprehensive anatomical evaluation and measurement of nerve in all sections (a) Horizontal slice; (b) nerve identification in 3D view

Corresponding locations on the mandibular ridge crest were identified to quantify linear distances between these anatomical landmarks. Vertical lines were drawn from the GT (as transferred from other sections), mental foramen, anterior-most aspect of AL, and 2 mm mesial to the loop, and the distance was calculated from all these lines to the GT (Figures 3, 4).

**Figure 5:**
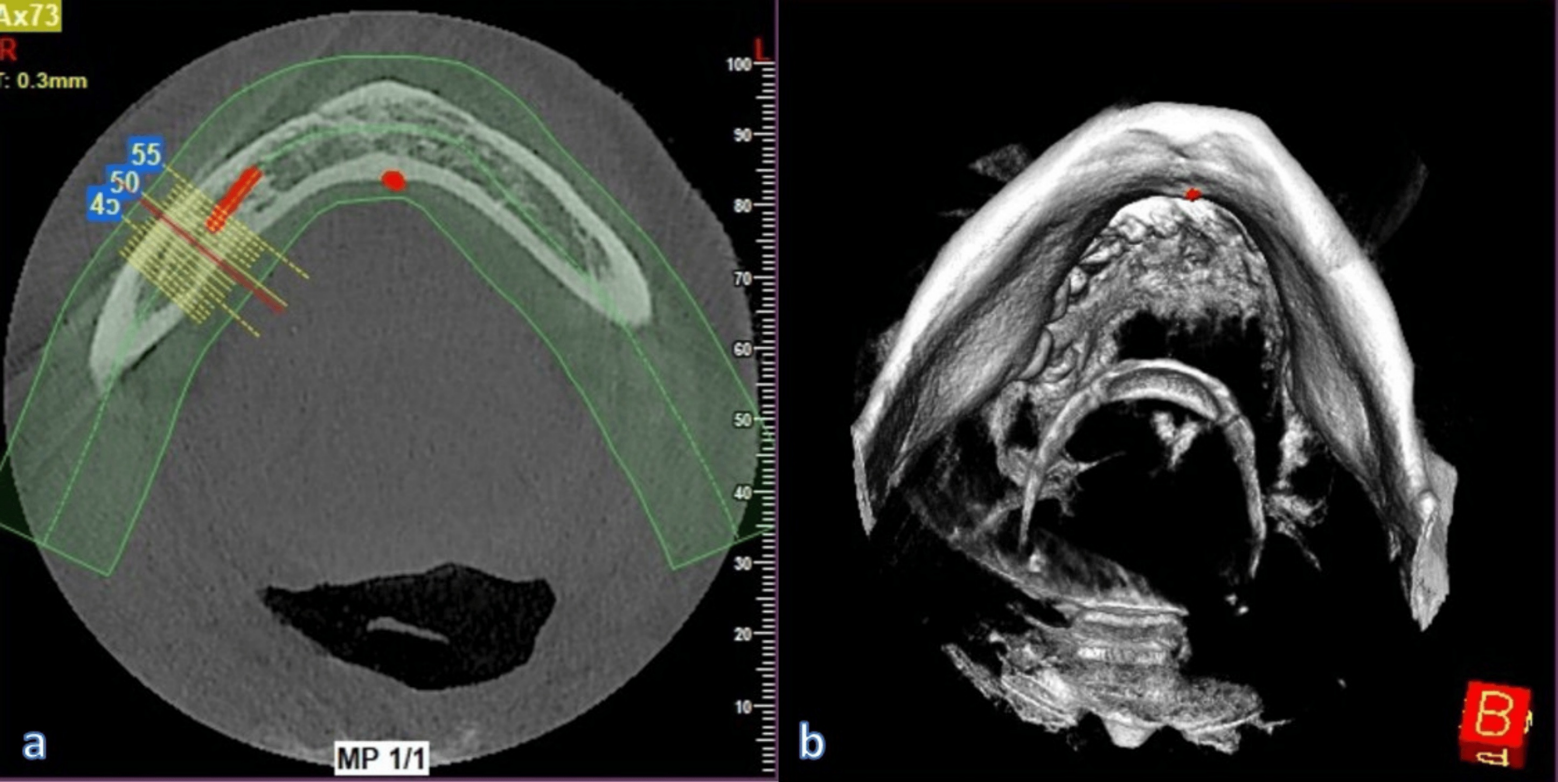
Determination of the location of the genial tubercle (a) In horizontal section; (b) in 3D view

**Figure 6:**
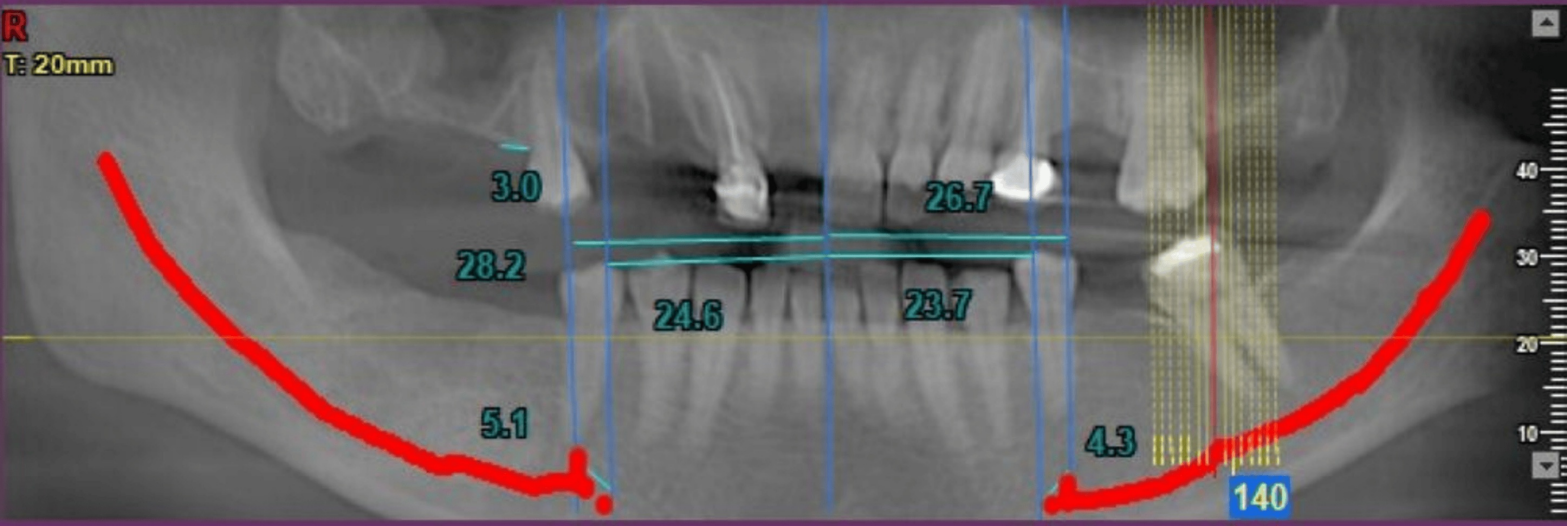
Measurements made in panoramic view

**Figure 7:**
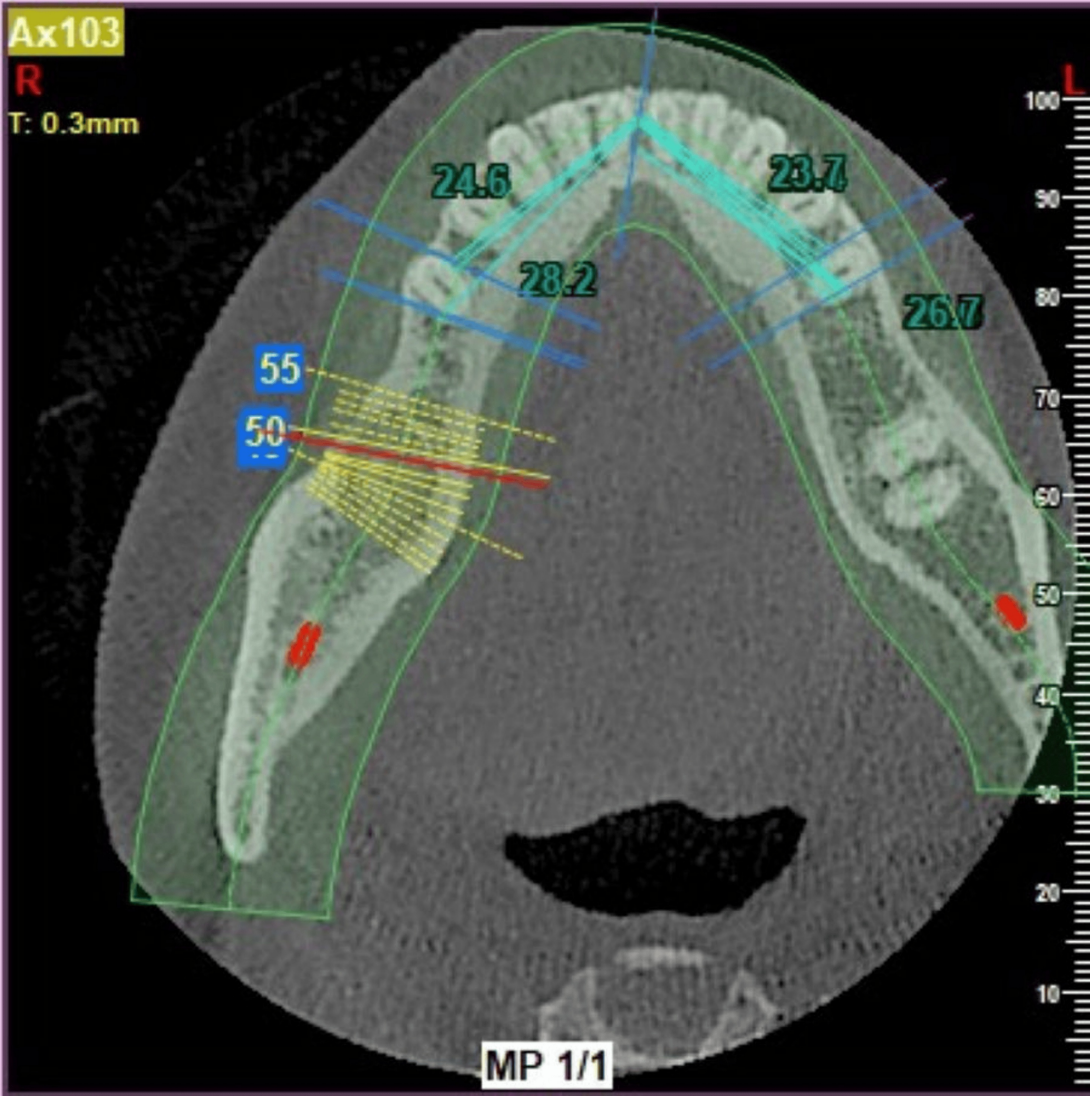
Measurements made in horizontal view

**Figure 8:**
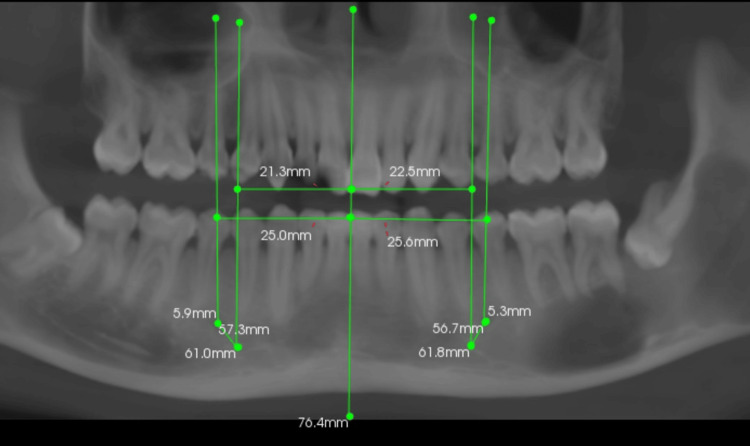
Panoramic measurements of other patients

**Figure 9:**
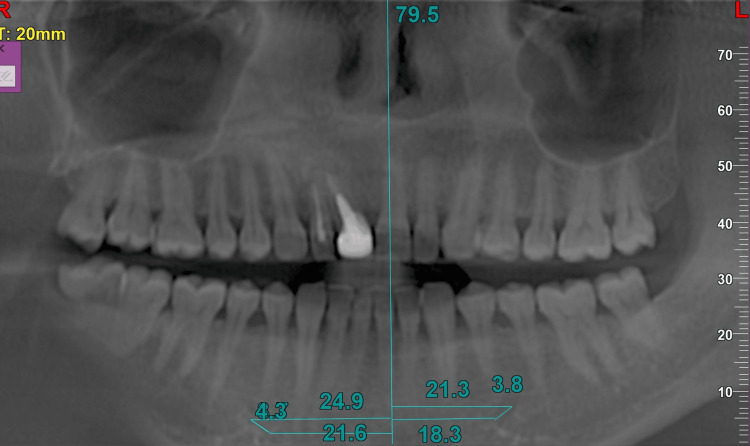
Panoramic measurements of other patients

The entire measurement process was then duplicated on the contralateral side. To define the safe zone, a 2 mm reduction was applied to the total distance between the GT and the AL, independently for both the left and right sides. Details of the measurements are mentioned in the following diagram. The measurements were cross-verified in the horizontal section (Figures 5-9).

Statistical Analysis

Mean distribution was calculated (Table 1) followed by a t-test, Tukey honestly significant difference (HSD), and linear regression analysis.

**Table 1:** Mean distribution of genial tubercle (GT) to mental foramen distance, GT to anterior loop distance, and safe zone in the right and left sides in men, women, and overall MIN: minimum; MAX: maximum; SD: standard deviation; SEM: standard error of the mean

Anatomic landmarks	Men						Women						Overall					
	MIN	MAX	Mean	SD	Median	SEM	MIN	MAX	Mean	SD	Median	SEM	MIN	MAX	Mean	SD	Median	SEM
Right GT to foramen	18.00	42.30	27.46	2.51	27.60	0.16	21.10	35.40	24.98	1.98	24.80	0.13	18.00	42.30	26.22	2.58	26.00	0.12
Left GT to foramen	19.00	36.80	26.28	2.37	26.50	0.15	18.30	32.40	23.52	2.05	23.40	0.13	18.30	36.80	24.90	2.61	24.80	0.12
Right GT-anterior loop	14.40	35.20	25.22	2.68	25.40	0.17	18.10	30.90	22.82	2.00	22.70	0.13	14.40	35.20	24.03	2.65	23.80	0.12
Left GT-anterior loop	15.90	33.30	24.07	2.53	24.30	0.16	14.70	27.80	21.46	2.10	21.50	0.13	14.70	33.30	22.77	2.66	22.80	0.12
Right safe zone (2 mm)	12.40	33.20	23.14	3.01	23.40	0.19	16.10	28.90	20.82	2.00	20.70	0.13	12.40	33.20	21.99	2.81	21.80	0.13
Left safe zone (2 mm)	13.90	31.30	22.07	2.54	22.30	0.16	12.70	26.80	19.47	2.12	19.50	0.13	12.70	31.30	20.77	2.67	20.80	0.12

## Results

GT to mental foramen distance (GT-foramen)

**Figure 10:**
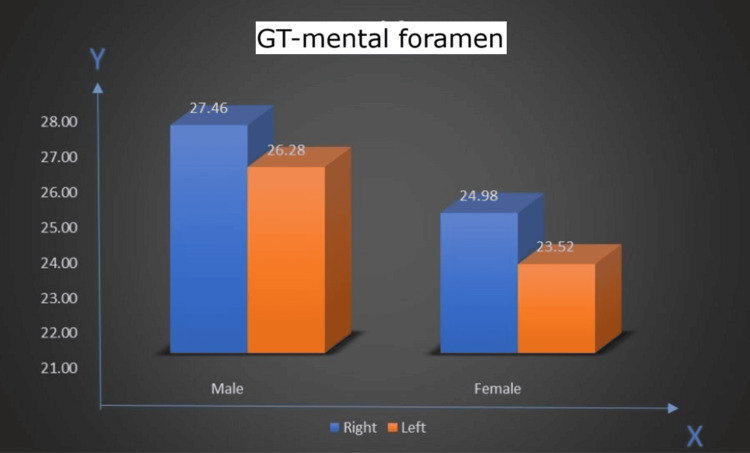
Distance between genial tubercle (GT) to mental foramen

In men, the distance between the right GT to mental foramen measured 27.46 mm on average, slightly greater than the left side at 26.28 mm. In women, the distance between the right GT to mental foramen was smaller at 24.98 mm, with the left side at 23.52 mm. The general distribution indicates that men had greater GT to mental foramen measurements compared to women (Figure 10).

GT to AL distance (GT-AL)

**Figure 11:**
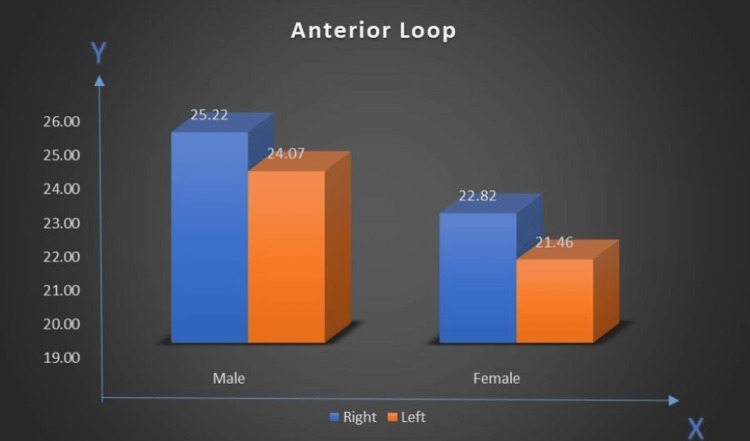
Distance between genial tubercle and anterior loop

Men averaged a right-side value of 25.22 mm and a left-side value of 24.07 mm. The corresponding values for women were 22.82 mm (right) and 21.46 mm (left). The measurement shows a larger distance in men compared to women (Figure 11).

Safe zone (2 mm)

**Figure 12:**
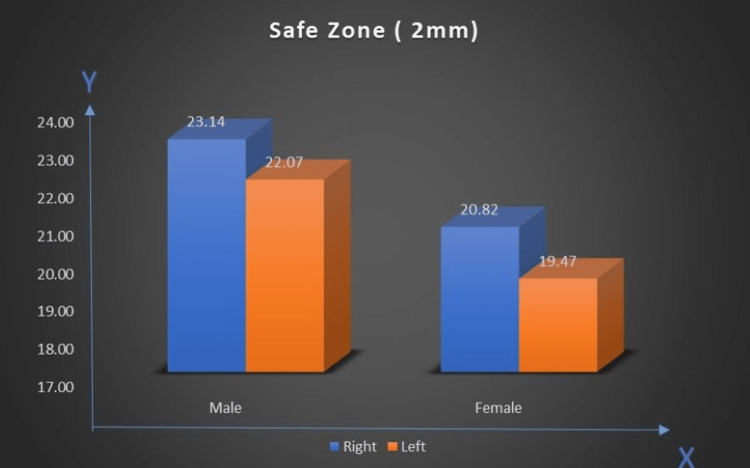
Comparison of safe zones

The average right-side safe zone for men was 23.14 mm, while on the left side, it was 22.07 mm. For women, the safe zone was smaller and had average measures of 20.82 mm (right) and 19.47 mm (left). The safe zone tended to be larger in men than in women (Figure 12).

Comparison between the right and left sides in male participants

A paired t-test was used to compare the right and left sides of every parameter in male subjects. The right-side GT-mental foramen was significantly larger than the left (p = 0.001). The GT-AL distance and safe zone were also significantly different between the right and left sides (p = 0.001), with all parameters being greater on the right (Table 2).

**Table 2:** Comparison of GT-mental foramen distance, GT-anterior loop distance, and safe zone (2 mm) on the right and left sides in male participants GT: genial tubercle; N: number of participants; t: test statistic; p-value: probability value

Anatomical landmarks	Side	N	Mean	Std. deviation	Std. error mean	Mean difference	t	p-value
GT-mental foramen	Right	250	27.4608	2.50730	0.15858	1.18480	18.481	0.001
	Left	250	26.2760	2.37371	0.15013			
GT-anterior loop	Right	250	25.2204	2.68043	0.16953	1.15000	18.328	0.001
	Left	250	24.0704	2.52877	0.15993			
Safe zone (2 mm)	Right	250	23.1408	3.01256	0.19053	1.07040	10.804	0.001
	Left	250	22.0704	2.53734	0.16048			

Comparison between the right and left sides in female participants

Comparable trends were noted in female subjects: The right-sided GT-mental foramen (24.98 mm) was greater than the left (23.52 mm), with a mean difference of 1.45 mm (p < 0.001). The GT-AL distance and safe zone also showed significant right-side dominance (p < 0.001) (Table 3).

**Table 3:** Comparison of GT-mental foramen distance, GT-anterior loop distance, and safe zone (2 mm) on the right and left sides in female participants GT: genial tubercle; N: number of participants; t: test statistic; p-value: probability value; Std.: standard

Anatomical landmarks	Side	N	Mean	Std. deviation	Std. error mean	Mean difference	t	p-value
GT-mental foramen	Right	249	24.9775	1.97875	0.12540	1.45462	22.306	0.000
	Left	249	23.5229	2.04917	0.12986			
GT-anterior loop	Right	249	22.8249	1.99956	0.12672	1.36104	21.703	0.000
	Left	249	21.4639	2.10276	0.13326			
Safe zone (2 mm)	Right	249	20.8249	1.99956	0.12672	1.35863	21.706	0.000
	Left	249	19.4663	2.11540	0.13406			

Comparison between women and men

A comparison between men and women shows men had a considerably higher value on all the parameters: men’s GT-foramen measured larger compared to women’s by around 2.48 mm (right) and 2.75 mm (left) (p < 0.001). The GT-AL distance and safe zone were also significantly larger in men compared to women (p < 0.001) (Table 4).

**Table 4:** Overall comparison of the GT-mental foramen distance, GT-anterior loop distance, and safe zone (2 mm) on the right and left sides between male and female participants GT: genial tubercle; N: number of participants; p-value: probability value

Gender and side		N	Minimum	Maximum	Mean	Std. deviation	F	p-value
GT-mental foramen	Male right	250	18.00	42.30	27.4608	2.50730	142.971	0.000
	Male left	250	19.00	36.80	26.2760	2.37371		
	Female right	249	21.10	35.40	24.9775	1.97875		
	Female left	249	18.30	32.40	23.5229	2.04917		
GT-anterior loop	Male right	250	14.40	35.20	25.2204	2.68043	118.529	0.000
	Male left	250	15.90	33.30	24.0704	2.52877		
	Female right	249	18.10	30.90	22.8249	1.99956		
	Female left	249	14.70	27.80	21.4639	2.10276		
Safe zone (2 mm)	Male right	250	12.40	33.20	23.1408	3.01256	104.603	0.000
	Male left	250	13.90	31.30	22.0704	2.53734		
	Female right	249	16.10	28.90	20.8249	1.99956		
	Female left	249	12.70	26.80	19.4663	2.11540		

Comparison of all groups

An ANOVA test established statistically significant differences in male and female participants for all parameters (p < 0.001). The largest GT-mental foramen, distance between GT-AL, and safe zone were found in men in all comparisons (Table 5).

**Table 5:** Overall, pairwise comparison of the anterior loop length, GT-mental foramen distance, GT-anterior loop distance, and safe zone (2 mm) on the right and left sides between male and female participants GT: genial tubercle; HSD: honestly significant difference

Multiple comparisons							
Tukey HSD							
Dependent variable	(I) Groups	(J) Groups	Mean difference (I-J)	Std. error	p-value	95% confidence interval	
						Lower bound	Upper bound
GT-mental foramen	Male right	Male left	1.18480	0.20022	0.000	0.6696	1.7000
		Female right	2.48329	0.20042	0.000	1.9675	2.9990
		Female left	3.93791	0.20042	0.000	3.4222	4.4537
	Male left	Female right	1.29849	0.20042	0.000	0.7827	1.8142
		Female left	2.75311	0.20042	0.000	2.2374	3.2689
	Female right	Female left	1.45462	0.20062	0.000	0.9384	1.9709
GT-anterior loop	Male right	Male left	1.15000	0.20981	0.000	0.6101	1.6899
		Female right	2.39550	0.21002	0.000	1.8550	2.9360
		Female left	3.75654	0.21002	0.000	3.2161	4.2970
	Male left	Female right	1.24550	0.21002	0.000	0.7050	1.7860
		Female left	2.60654	0.21002	0.000	2.0661	3.1470
	Female right	Female left	1.36104	0.21023	0.000	0.8200	1.9020
Safe zone (2 mm)	Male right	Male left	1.07040	0.21909	0.000	0.5066	1.6342
		Female right	2.31590	0.21931	0.000	1.7515	2.8803
		Female left	3.67453	0.21931	0.000	3.1102	4.2389
	Male left	Female right	1.24550	0.21931	0.000	0.6811	1.8099
		Female left	2.60413	0.21931	0.000	2.0398	3.1685
	Female right	Female left	1.35863	0.21953	0.000	0.7937	1.9236

Safe zone categorization in men and women

A categoric breakdown of the safe zone revealed that a greater proportion of women (34.1%) belonged to category 2, i.e., 15-19.9 mm category, than men (9.2%) (Table 6). Most participants (66.3%) were in category 3, i.e., 20-24.9 mm category. Men tend to have a safe zone > 25 mm (20.8%) more than women (2.4%), i.e., category 4. There was a statistically significant difference (p = 0.001), which implies that men have a wider safe zone than women.

**Table 6:** Categorization of safe zones n: number of participants

Categories based on calculated safe zones			Gender		Total	p-value
			Male	Female		
Categorization	Category 1: 10-14.9 mm	n	2	0	2	0.001
		%	0.8%	0.0%	0.4%	
	Category 2: 15-19.9 mm	n	23	85	108	
		%	9.2%	34.1%	21.6%	
	Category 3: 20-24.9 mm	n	173	158	331	
		%	69.2%	63.5%	66.3%	
	Category 4: >25 mm	n	52	6	58	
		%	20.8%	2.4%	11.6%	
Total		n	250	249	499	
		%	100.0%	100.0%	100.0%	

Linear regression equation showing the predictors of safe zones for the right side

The linear regression equation shows an excellent fit to the data, reflected by an R of 0.948 and an R² of 0.899 (Table 7). This indicates that the predictors in the model explain about 89.9% of the variance in the dependent variable. Looking at the model coefficients, the intercept is calculated at -2.49864 with a standard error (SE) of 0.5783 and is statistically significant (p < 0.001). Gender is not a significant predictor since the difference between women and men (0.11946, SE = 0.1126) is not statistically significant (p = 0.289). Nevertheless, right GT-AL is a strong and statistically significant predictor with an estimate of 0.99894 (SE = 0.0529, p < 0.001), suggesting that it plays a significant role in the dependent variable. In general, all predictors are not significantly contributing to the model except for right GT-AL, which has a vital contribution in explaining the variance of the outcome.

**Table 7:** Regression depicting predictors of safe zones on the right side SE: standard error; t: test statistic; p: probability value; GT: genial tubercle ᵃRepresents reference level

Model coefficients-right safe zone (2 mm)				
Predictor	Estimate	SE	t	p
Interceptᵃ	-2.49864	0.5783	-4.3209	
Female-male	0.11946	0.1126	1.0605	0.289
Right GT-mental foramen	0.01533	0.0565	0.2713	0.786
Right GT-anterior loop	0.99894	0.0529	18.8781	

Linear regression analysis of the predictors of the safe zone for the left side

The linear regression model gives a good fit to the data with an R of 1.000 and an R² of 0.999 (Table 8). This means that the model accounts for almost all the variance in the dependent variable. Looking at the model coefficients, the intercept is estimated at -2.11746 with a SE of 0.04737, and it is very significant (p < 0.001). The gender (women-men) predictor is estimated as 0.01435 (SE = 0.00951), yet is not significant statistically (p = 0.132), and this implies gender does not make a significant contribution to the result. Left GT-AL is a very powerful predictor with an estimate of 0.99889 (SE = 0.00447, p < 0.001). This suggests that it has a very strong effect on the outcome variable. In general, most predictors in the model are not statistically significant, but left GT-AL contributes very strongly to explaining variance in the dependent variable.

**Table 8:** Regression depicting predictors of the safe zone on the left side SE: standard error; t: test statistic; p: probability value; GT: genial tubercle ᵃRepresents reference level

Model coefficients-left safe zone (2 mm)				
Predictor	Estimate	SE	t	p
Interceptᵃ	-2.11746	0.04737	-44.698	
Female-male	0.01435	0.00951	1.508	0.132
Left GT-mental foramen	0.00564	0.00475	1.187	0.236
Left GT-anterior loop	0.99889	0.00447	223.528	

## Discussion

Accurate knowledge of anatomical structures in the anterior mandible is crucial to avoid neurovascular complications during implant placement. Among these, the AL of the IAN and the GT are especially important. However, both structures exhibit considerable anatomical variability across populations, underscoring the need for population-specific assessments.

In the present study, the prevalence and dimensions of the AL and the distance between the GT and mental foramen were evaluated using CBCT. The prevalence of AL varies widely across the literature. Soman et al. [8] reported a 4.24% prevalence in a Saudi cohort, while Mukherjee et al. [9] and Jena et al. [10] found a much higher prevalence (72%) in Odisha. Our study aligns more closely with the latter, reinforcing the notion that regional anatomical differences are significant.

The GT, another critical landmark, also exhibited gender-based differences. The mean distance from the GT to the AL was 25.22 mm (right) and 24.07 mm (left) in men, compared to 22.82 and 21.46 mm in women. These findings exceed those reported by Voon and Patil [6], again underscoring regional variation.

After subtracting a 2 mm safety margin from the GT-to-AL distance, a “safe zone” for implant osteotomy was calculated. These safe zones were significantly larger in men (23.14 mm right; 21.92 mm left) than in women (20.72 mm right; 19.34 mm left). These values suggest a more conservative approach may be warranted in female patients or those with smaller mandibular dimensions.

Categorization of the safe zone based on the findings

Based on the results of the study conducted, the safe zone was divided into four categories: category 1: 10 to 14.9 mm, category 2: 15 to 19.9 mm, category 3: 20 to 24.9 mm, and category 4: >25 mm. Most individuals (66.3%) fell within category 3 (20-24.9 mm). A significantly higher proportion of women were in category 2 (15-19.9 mm), while a notable percentage of men were in category 4 (>25 mm), demonstrating statistically significant sex-based variation (p = 0.001).

The role of CBCT in detecting these anatomical structures cannot be overstated. Several studies, including de Oliveira-Santos et al. [11] and Pires et al. [12], have shown CBCT to be superior to panoramic radiography in detecting the mental canal and incisive canal, with detection rates up to 83%. Mardinger et al. [13] emphasized the limited reliability of panoramic imaging in representing true neural anatomy.

These findings have direct clinical relevance. Authors Xu et al. [14] and Sener et al. [15] recommend maintaining at least 2-4 mm of clearance from vital structures during osteotomy. Additionally, Vyas and Tadinada [16] emphasized the need to identify the sublingual artery to prevent intraoperative hemorrhage. Preoperative CBCT not only enables these precautions but also facilitates digital planning and surgical guides, improving safety and predictability. Pedrinaci et al. [17] advocated for such computer-assisted approaches in implant dentistry, especially in complicated cases. Koenig et al. [18] demonstrated the value of virtual surgical planning (VSP) in complex mandibular trauma, illustrating how CBCT-based workflows can be adapted for precision surgical guide fabrication.

In summary, this study reinforces the importance of CBCT-based morphometric evaluation of the anterior mandible. Given the significant anatomical variability based on sex, side, and population, preoperative imaging and individualized planning are essential for safe and successful implant placement.

Limitations

The study population consisted of 499 participants, and the findings may not be generalizable to other populations. Also, the study relied on CBCT measurements, which may have inherent limitations in accuracy.

Future directions

Further research is needed to investigate the long-term outcomes of implant procedures in relation to gender-based anatomical variations. Future studies could explore the use of advanced imaging techniques and computer-assisted implant surgery to optimize implant outcomes. In conclusion, this study provides valuable insights into the gender-based differences in mandibular morphology and their clinical implications for implant dentistry. The findings underscore the importance of meticulous preoperative evaluation and consideration of patient-specific factors in implant planning and surgical procedures.

## Conclusions

The gender variations of mandibular morphology as seen have significant clinical implications for implant planning. A thorough assessment of individual anatomy using CBCT is critical to prevent complications such as mandibular incisive canal (MIC) perforation and nerve injury. The study emphasizes the importance of patient-specific factors being considered in implant planning and surgery. For instance, the wider safe zone in men can allow greater flexibility in implant placement.
